# Pathway Databases

**DOI:** 10.1002/cfg.123

**Published:** 2001-12

**Authors:** Jo Wixon

**Affiliations:** Bioinformatics Division HGMP-RC Hinxton, Cambridge CB10 1SB UK


**Comparative and Functional Genomics**
*Comp Funct Genom* 2001; **2**: 391–397.
**DOI:** 10.1002/cfg.123

**Website Review**

### Pathway databases

Jo Wixon, Managing Editor
*Bioinformatics Division, HGMP-RC, Hinxton, Cambridge, CB10 1SB, UK*

Published online:
20 November 2001

### Introduction

Following on from the profile of web resources for protein-protein interactions in our last issue, we present a survey of pathway databases. The resources available have, in the past, been mainly concerned with metabolic pathways, which are in general, better characterised. However, the number of resources featuring regulatory pathways, or networks, has been increasing for some time.

This area has great potential to evolve towards a more complex understanding of the networks in operation inside cells, sometimes referred to as systems (or integrative) biology, but this will require databases that encode the underlying theories and allow the integration of other datasets, such as protein and mRNA profiling results, rather than simple graphical representations of pathways. Eventually, this could lead to the ability to predict flux through pathways, and the effects of knocking out a gene in a network.

### Metabolic and other pathways

### What is there? (WIT)

**http://wit.mcs.anl.gov/WIT2/**

This extensive resource is described as 'interactive metabolic reconstruction on the web', but also includes data on transport and signal transduction pathways (Overbeek *et al.*, 2000). It uses data from EMP (see below) to provide metabolic reconstructions for sequenced (or partially sequenced) genomes, and covers 39 genomes at the moment (seven archaea, 30 bacteria and two eukaryota). The data can be queried and visualised in several ways, including 'functional overviews', which are hierarchical listings of the pathways present in a chosen organism, 'ORF pages', which provide details and links to more information on a protein of interest, and a listing of the proteins most similar to it, and 'pathway pages', which provide a list of the proteins involved in a pathway, and whether they have been identified in an organism of choice, followed by an assertions table indicating the presence or absence of the pathway members in the 39 genomes. A pathway query leads to a shortened pathway page (Figure 1) without the assertions table (this can be activated if required). The pathway can also be viewed as a diagram (Figure 2) and a table of the data held on that pathway is also made available.

### Kyoto Encyclopaedia of Genes and Genomes (KEGG)

**http://www.genome.ad.jp/kegg/**

This website provides graphical representations of metabolic and regulatory pathways in a wide range of organisms, and also maintains a catalogue of completed and ongoing genome sequencing projects (Kanehisa and Goto, 2000). This extensive site has been reviewed in detail, in the first issue of CFG (Wixon and Kell, 2000).

### MetaCyc and EcoCyc

**http://ecocyc.org**

The EcoCyc database of *E. coli* metabolic, transport and genetic regulatory networks is structured according to an ontology and consists of a web of frames (objects, eg. genes) stored in a frame knowledge representation system (Karp, 2001). Connections between the frames represent relationships between the objects. Reasoning across this

Copyright © 2001 John Wiley & Sons, Ltd.

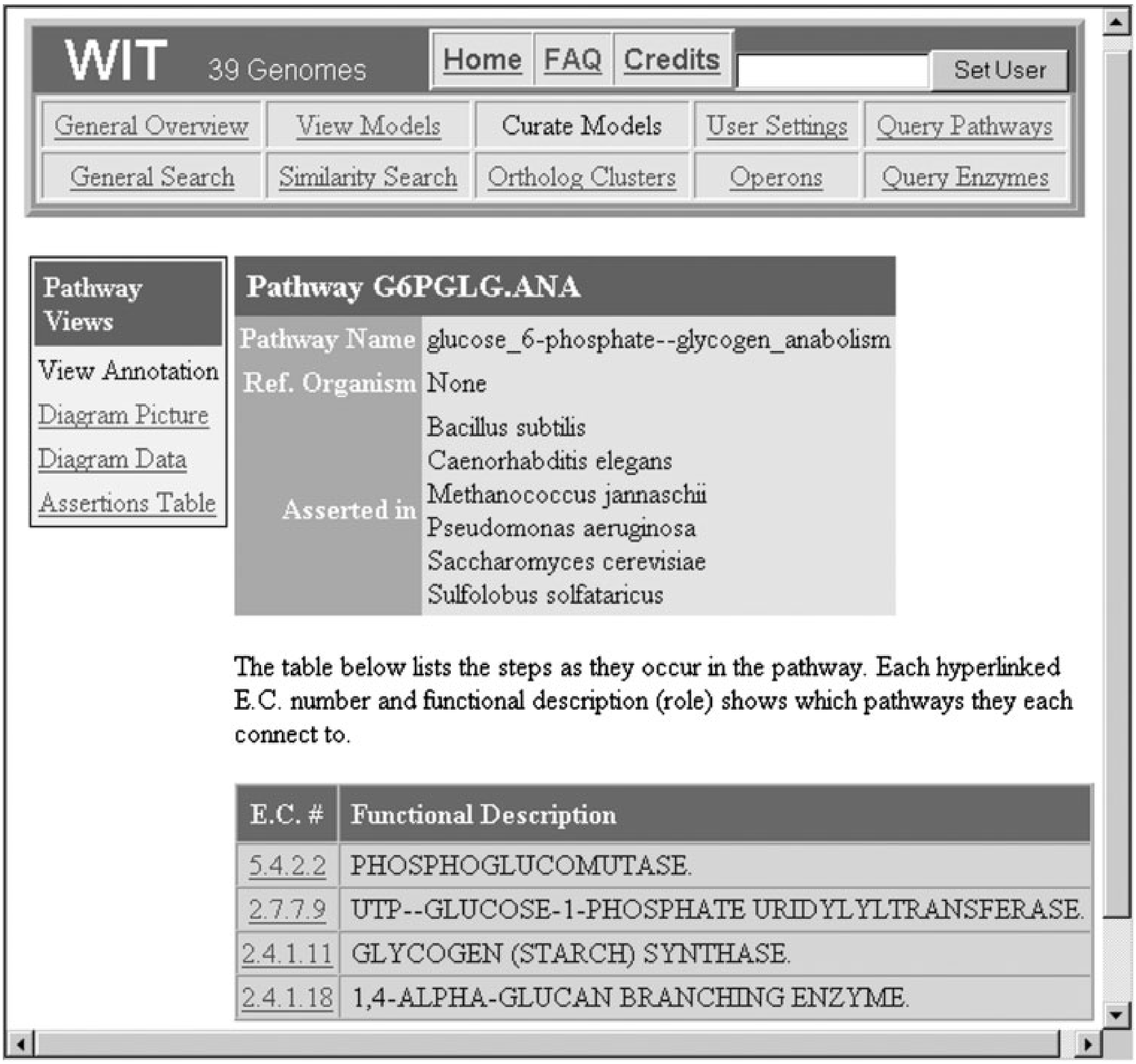

**Figure 1.** The pathway in WIT for glucose-6-phosphate to glycogen anabolism. Organisms which have this pathway and those in which it has been asserted are listed in the upper table, the enzymes forming the pathway are listed in the lower table. This image is reproduced by kind permission of R. Overbeek and E. Selkov, Jr. © US Government database is accomplished by symbolic (non-numerical) computing. This allows global analyses, such as investigations into how many steps are catalysed by multiple enzymes (ie. potentially redundant), or how interconnected the members of the network are. The *E.coli* metabolic overview diagram shows reactions as coloured lines, between shapes representing metabolites (the particular shape of each metabolite denotes its chemical class). Those reactions not yet assigned to a pathway are represented on the right hand side of the diagram, the network is shown on the left. The network can be compared to that of another species, with the conserved and non-conserved reactions being picked out in different colours. EcoCyc has also been used to predict the metabolic network of *Haemophilus influenzae* from its genome (Karp *et al.*, 1996), and there are similar resources for a range of other bacteria and yeast. These resources, and MetaCyc (a collection of the pathways across all of these organisms, with other data obtained from the literature), can be queried by

Copyright © 2001 John Wiley & Sons, Ltd. 

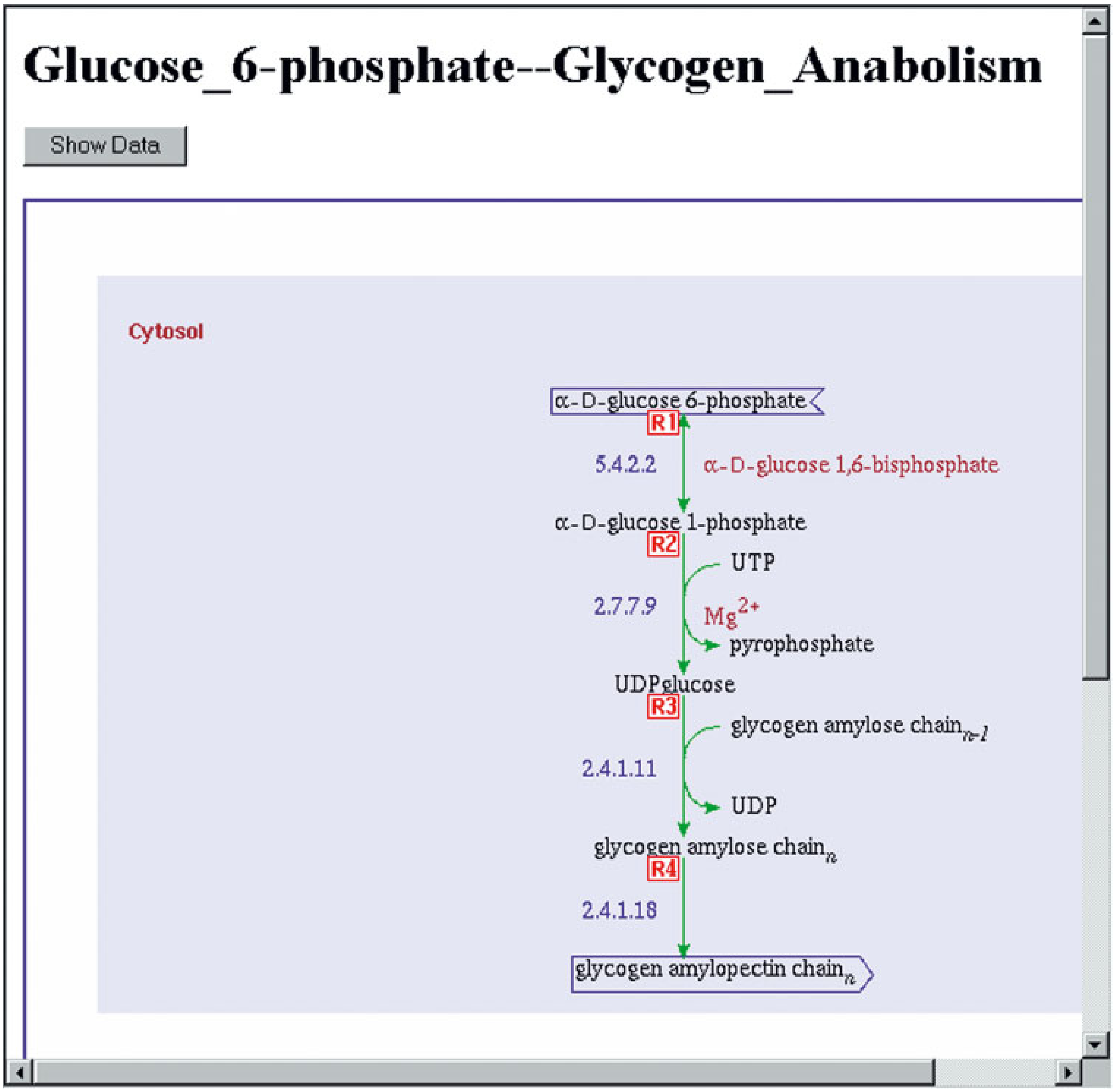

**Figure 2.** The WIT pathway diagram for glucose-6-phosphate to glycogen anabolism. Enzymes are shown in blue and reactants and products in black. Clicking on an enzyme or a compound leads to further information on that component of the pathway. This image is reproduced by kind permission of R. Overbeek and E. Selkov, Jr. © US Government name or EC number, or browsed by a selection of classification hierarchies (**http://ecocyc.org:1555/server.html?**).

### BioCarta

**http://www.biocarta.com**

This commercial site is home to the Proteomic Pathway Project (P3), an 'open source, community-led' project to accumulate, and represent online, current knowledge on pathways. Users can browse the entire pathway set, which is grouped into categories ranging from adhesion to neuroscience. Some of the pathways have been contributed by academics, the others have been entered by BioCarta. The home page also has quick links to featured pathways and genes, apparently selected based on current research trends. Clicking on a protein in a pathway diagram leads to a page with links out to external database entries relating to known orthologs of that gene, the IL-4 page, for example, has links to information on human, mouse and rat IL-4, from a wide range of databases.

### Protein Function and Biochemical Pathways (PFBP) Project

**http://www.ebi.ac.uk/research/pfbp/**

A research group at the EBI has formed a consortium with Hoechst Marion Roussel, AstraZeneca (Pharma division), Monsanto (Cereon), Organon (Akzo-Nobel) and Roche, to develop a European database for information on Protein Function and Metabolic Pathways. The result is the aMAZE database (van Helden *et al.*, 2000); prototype tools for searching this resource are available at the site.

### Biological Biochemical Image Database (BBID)

**http://bbid.grc.nia.nih.gov/**

This database is a collection of images, obtained from the literature, of biological pathways, macromolecular structures, gene families and cellular relationships (Becker *et al.*, 2000). It can be searched using keywords such as gene names, pathway names and cell or tissue types.

### Metabolic pathways only

### Enzymes and Metabolic Pathways Database (EMP)

**http://emp.mcs.anl.gov/**

This site holds a vast array of information on metabolic pathways, reaction mechanisms and numerical data, such as rate laws. There are also entries for transporters. There are currently around 3000 pathway diagrams and 30 000 records. The database is constructed by a team of researchers, using information obtained from the published literature. The aim is to translate the factual content of original journal articles into a structured, indexed, and easily searchable form. The EMP Pathways resource was formerly known as the Metabolic Pathways Database (MPW, Selkov *et al.*, 1998) and can be queried separately from the rest of the EMP database.

### Microbial Biocatalysis and Biodegradation UM-BBD

**http://umbbd.ahc.umn.edu/**

This database contains information on microbial biocatalytic reactions and biodegradation pathways (primarily for xenobiotic, chemical compounds). The goal of the UM-BBD is to provide information on microbial enzyme-catalysed reactions that are important for biotechnology (Ellis *et al.*, 2001).

### Soybean Metabolism (SoyBase Metabolic Component)

**http://cgsc.biology.yale.edu/metab.html**

This subsection of SoyBase (which is an ACEDB database for the soybean genome) has diagrams and reaction and pathway descriptions for a number of basic metabolic pathways. The data is made available on the Web using a translation program, which is still under development.

### Regulatory and signaling pathways

### TRANSPATH Signal Transduction Browser

**http://transpath.gbf.de/**

TRANSPATH provides information, obtained from the scientific literature, on gene-regulatory pathways, mainly those of human, mouse and rat (Heinemeyer *et al.*, 1999). It is concerned primarily with pathways involved in the regulation of transcription factors. The object-oriented database and holds information on hormones, enzymes, complexes, transcription factors, their interactions, and references to the literature. Users can search the resource using keywords against various fields, such as molecule, or reaction, or browse the pathway maps. These maps illustrate the subcellular locations of the proteins involved, the nature of their interactions, and any protein modifications, such as phosphorylation or ubiquitination.

### Signaling PAthway Database (SPAD)

**http://www.grt.kyushu-u.ac.jp/eny-doc/**

The pathways are categorised by the extracellular signalling molecules that initiate them (Growth Factor, Cytokine, Hormone and Stress). The pathway maps show the subcellular locations of the protein components and use their shape to differentiate between active and inactive forms and their colour to indicate the class of protein (eg.

Copyright © 2001 John Wiley & Sons, Ltd.

plasma membrane receptor, or transcription factor). Clicking on a protein leads to a page with its GenBank entry and an option to search Medline or SWISS-PROT with the protein name as a keyword.

### Cell Signaling Networks DataBase (CSNDB)

**http://geo.nihs.go.jp/csndb/**

This database holds signalling pathways for human cells, with information on the biological molecules, sequences, structures, functions, and biological reactions involved in transferring cellular signals (Takai-Igarashi *et al.*, 1998), that has been collected from published literature. The resource can be browsed by molecule type, or queried, using a global search or an ACEdb query, or using the pathway finder (Takai-Igarashi and Kaminuma, 1999). The pathways are compiled as binary relationships of biomolecules and represented by graphs, which are drawn automatically (Figure 3). If a network has more than one role, the option to colour those steps involved in a chosen role is available. Clicking on a protein in the pathway leads to its molecule page. These pages contain an array of information, including synonyms, chromosomal location, functional and expression information, and links to the pathways in which it is involved, sequence database entries and references.

### GeneNet

**http://wwwmgs.bionet.nsc.ru/mgs/systems/genenet**

GeneNet (Kolpakov *et al.*, 1998) can be accessed by making an SRS query, or by activating the

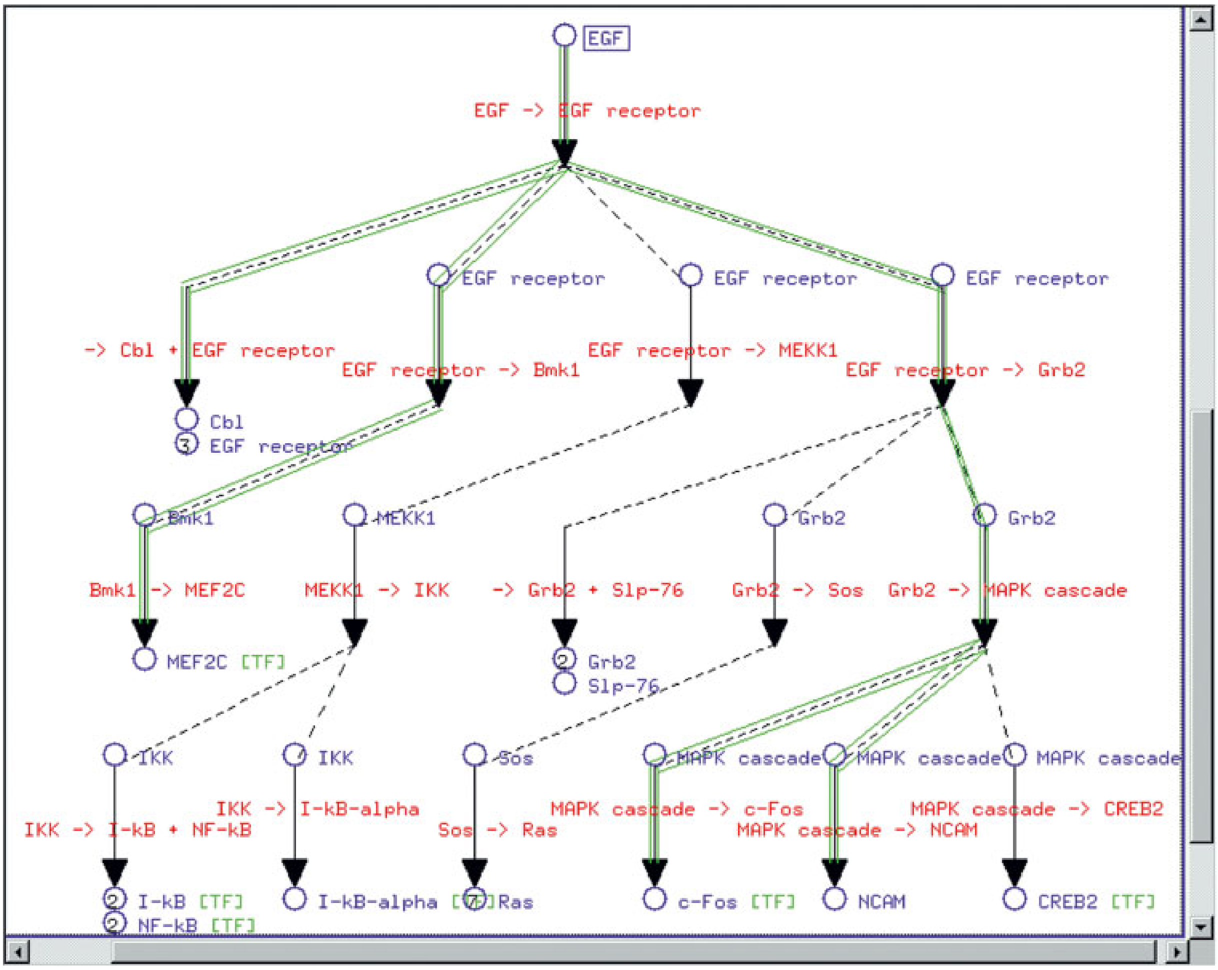

**Figure 3.** The EGF pathway, to 4 steps depth, from CSNDB. Proteins are named in blue, transcription factors are marked with [TF] in green. Those steps of the pathway involved in cell growth signalling have been highlighted in green. This image is reproduced by kind permission of T. Takai-Igarashi, © T. Takai-Igarashi

Copyright © 2001 John Wiley & Sons, Ltd.

GeneNet viewer, a Java applet for visualising and exploring networks (this can take quite some time to load). The 23 networks currently available are arranged in seven sections: antiviral response, lipid metabolism, endocrine system, erythroid differentiation, plant gene networks, REDOX-regulation and heat shock response. Users can register for access via a data input graphical user interface (GUI) and there is also a modelling facility.

### The Science Signal Transduction Knowledge Environment (STKE) Connections Map

**http://stke.sciencemag.org/cm/**

The entries in this database have been contributed by named experts in each field. As yet the resource is limited in scope, but it contains a mix of canonical and organism/tissue specific pathways. The proteins in the pathways are linked by arrows, which show the nature of their relationship, and coloured to indicate their subcellular location (this is a rough guide, as several components are known to move location during signalling).

### Gene Networks Database (GeNet)

**http://www.csa.ru/Inst/gorb_dep/inbios/genet/genet.htm**

GeNet holds information on the functional organization of regulatory gene networks acting at embryogenesis (Serov *et al*., 1998). There are two areas of the resource; EmbryoNet, which contains genetic networks controlling development; and SSE, which will hold genetic networks controlling stress response in Eukaryotes (this is not yet available). EmbryoNet is made up of UrchiNet (Sea Urchin developmental networks), SegNet (*Drosophila* developmental networks and expression patterns) and VertNet (which currently only holds information on the Wnt gene family). The 'NetModel' pages have detailed information on three approaches for building models of genetic networks.

### Comprehensive Yeast Genome Database (Pathways Section)

**http://mips.gsf.de/proj/yeast/CYGD/db/index.html**

This pathways section of this resource is a collection of figures (some of which link the proteins to their full database entries) of pathways donated by yeast researchers. They are categorised into Carbohydrate metabolism, Lipid, fatty-acid, and sterol metabolism, Energy, Cell growth, cell division and DNA synthesis, Transcription, Protein destination, Intracellular transport and Signal transduction. This recently expanded resource was originally known as MIPS yeast (Munich Information centre for Protein Sequences, Mewes *et al*., 1997).

### Interactive Fly - *Drosophila* Genes

**http://sdb.bio.purdue.edu/fly/aimain/1aahome.htm**

This guide to *Drosophila melanogaster* genes and their roles in development includes developmental and biochemical pathways in the fly.

### Related articles

Burgard AP, Maranas CD. 2001. Probing the performance limits of the *Escherichia coli* metabolic network subject to gene additions or deletions. *Biotechnol Bioeng* **74**: 364–375.

Fukuda Ki, Takagi T. 2001. Knowledge representation of signal transduction pathways. *Bioinformatics* **17**: 829–837.

Wittig U, De Beuckelaer A. 2001. Analysis and comparison of metabolic pathway databases. *Brief Bioinform* **2**: 126–142.

Yaffe MB, Leparc GG, Lai J, Obata T, Volinia S, Cantley LC. 2001. A motif-based profile scanning approach for genome-wide prediction of signaling pathways. *Nat Biotechnol* **19**: 348–353.

### References

Becker KG, White SL, Muller J, Engel J. 2000. BBID: the biological biochemical image database. *Bioinformatics* **16**: 745–746.

Ellis LB, Hershberger CD, Bryan EM, Wackett LP. 2001. The University of Minnesota Biocatalysis/Biodegradation Database: emphasizing enzymes. *Nucleic Acids Res* **29**: 340–343.

Heinemeyer T, Chen X, Karas H, *et al*. 1999. Expanding the TRANSFAC database towards an expert system of regulatory molecular mechanisms. *Nucleic Acids Res* **27**: 318–322.

Kanehisa M, Goto S. 2000. KEGG: Kyoto encyclopedia of genes and genomes. *Nucleic Acids Res* **28**: 27–30.

Karp PD. 2001. Pathway databases: a case study in computational symbolic theories. *Science* **293**: 2040–2044.

Karp PD, Ouzounis C, Paley S. 1996. HinCyc: a knowledge base of the complete genome and metabolic pathways of *H. influenzae*. *Proc Int Conf Intell Syst Mol Biol* **4**: 116–124.

Kolpakov FA, Ananko EA, Kolesov GB, Kolchanov NA. 1998. GeneNet: a gene network database and its automated visualization. *Bioinformatics* **14**: 529–537.

Mewes HW, Albermann K, Heumann K, Liebl S, Pfeiffer F.

Copyright © 2001 John Wiley & Sons, Ltd. 

1997. MIPS: A database for protein sequences, homology data and yeast genome information. *Nucleic Acids Res* **25**: 28–30.
Overbeek R, Larsen N, Pusch GD, *et al.* 2000. WIT: integrated system for high-throughput genome sequence analysis and metabolic reconstruction. *Nucleic Acids Res* **28**: 123–125.
Selkov E Jr, Grechkin Y, Mikhailova N, Selkov E. 1998. MPW: the Metabolic Pathways Database. *Nucleic Acids Res* **26**: 43–45.
Serov VN, Spirov AV, Samsonova MG. 1998. Graphical interface to the genetic network database GeNet. *Bioinformatics* **14**: 546–547.
Takai-Igarashi T, Kaminuma T. 1999. A Pathway Finding System for the Cell Signaling Networks Database. *In Silico Biology* **1**: 129–146.
Takai-Igarashi T, Nadaoka Y, Kaminuma T. 1998. A database for cell signaling networks. *J Comp Biol* **5**: 747–754.
van Helden J, Naim A, Mancuso R, *et al.* 2000. Representing and analysing molecular and cellular function using the computer. *Biological Chemistry* **381**: 921–935.
Wixon J, Kell D. 2000. Website review – The Kyoto Encyclopedia of Genes and Genomes – KEGG. *Yeast* **17**: 48–55.

---

Some of the sites reviewed will already be known to you, but perhaps their content will be less well-known. The Website Review is intended to help you discover new sites of interest, but also to provide a rapid and convenient means of revealing what you always knew was there but never had the time or inclination to look at. These articles are a personal critical analysis of the Websites. If you have any information about sites you think are worthy of being more widely known, the Managing Editor would be pleased to hear from you.

The Genomics Website at Wiley www.wiley.co.uk/genomics

The Genomics website at Wiley is a DYNAMIC resource for the genomics community, offering FREE special feature articles and new information EACH MONTH.

Find out more about our new journals *Comparative and Functional Genomics,* and *Proteomics.*

Visit the Library for hot books in Genomics, Bioinformatics, Molecular Genetics and more.

Click on Journals for information on all our up-to-the minute journals, including: *Genesis, Bioessays, Journal of Mass Spectrometry, Gene Function and Disease, Human Mutation, Genes, Chromosomes and Cancer* and the *Journal of Gene Medicine.*

Let the Genomics website at Wiley be your guide to genomics-related web sites, manufacturers and suppliers, and a calendar of conferences.

WILEY

2380

Copyright © 2001 John Wiley & Sons, Ltd. 
